# Supplementary figures and images for: Multi-strain phage induced clearance of bacterial infections
Source: PLoS Comput Biol. 2025 Feb 4;21(2):e1012793. doi: 10.1371/journal.pcbi.1012793 (PMC11828373; doi:10.1371/journal.pcbi.1012793)

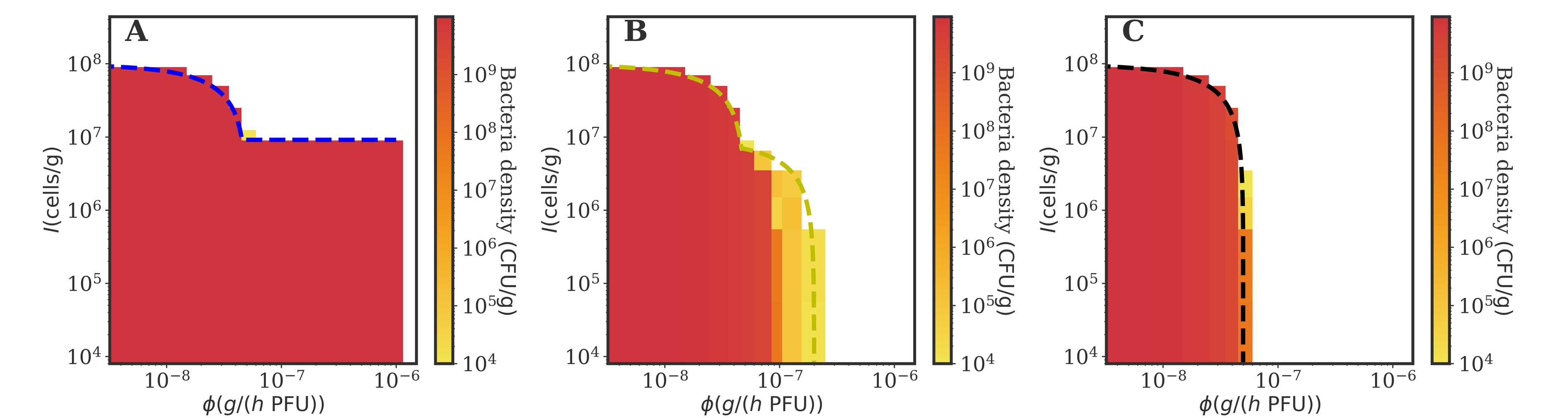

Supplement: S1 Fig — Numerical simulations of the model in Eq. (4) varying I and ϕ, with p = 0 (A), p = 0 . 5 (B) and without allowing for phage resistance (C). The colormap represents the density of bacteria in the last part of the numerical simulations. The dashed line represents the prediction in Eq. (11) for p = 0 in A) and 0 . 5 in B) (blue and yellow respectively). The black dashed line in C) shows Eq. (10), representing therapy success when bacteria do not develop phage resistance. Simulation parameters are reported in Table 2. (TIF) [file pcbi.1012793.s002.tif]

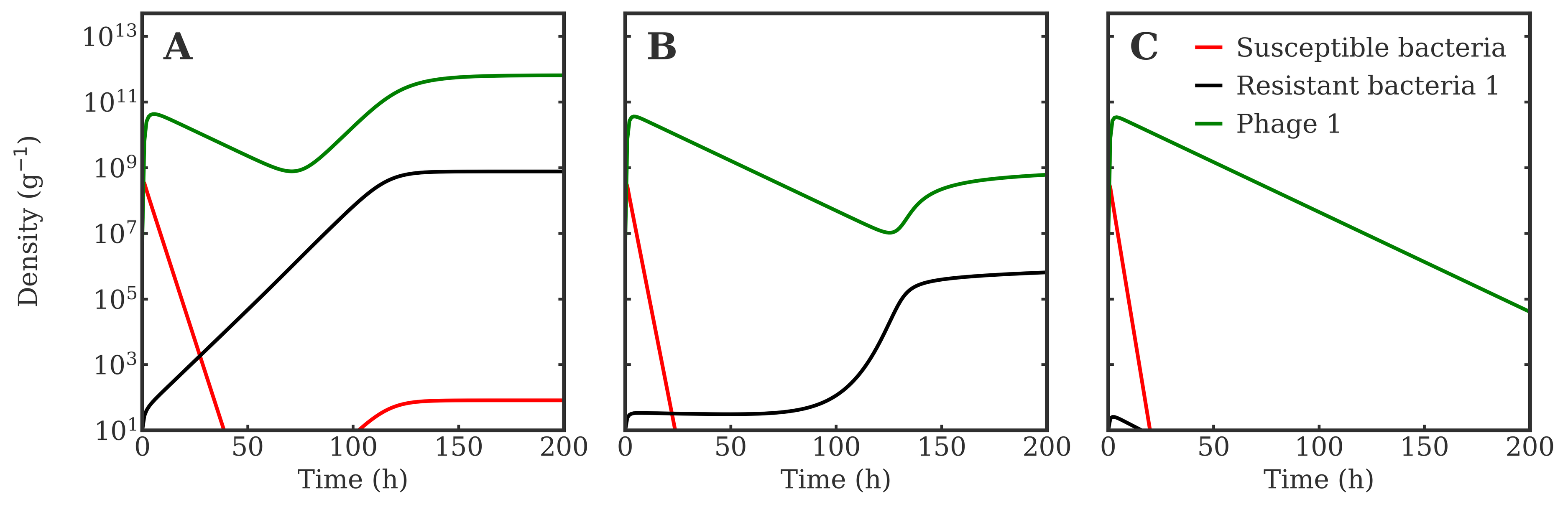

Supplement: S2 Fig — Numerical simulations of the model in Eq. (4), with p = 1 and I=105. Each panel shows the population dynamics for a different value of ϕ close to the sharp therapy success transition in Eq. (11). A) ϕ=8⋅10-8 falls within the red region of the phase diagram in Fig 6, with bacteria saturating to carrying capacity KC. B) With ϕ=10-7, right at the theoretical transition, bacteria coexist with phage at an intermediate density. C) Just above the transition at ϕ=1.1⋅10-7 bacteria are cleared, corresponding to the white region in the phase diagrams. The other simulation parameters are reported in Table 2. (TIF) [file pcbi.1012793.s003.tif]

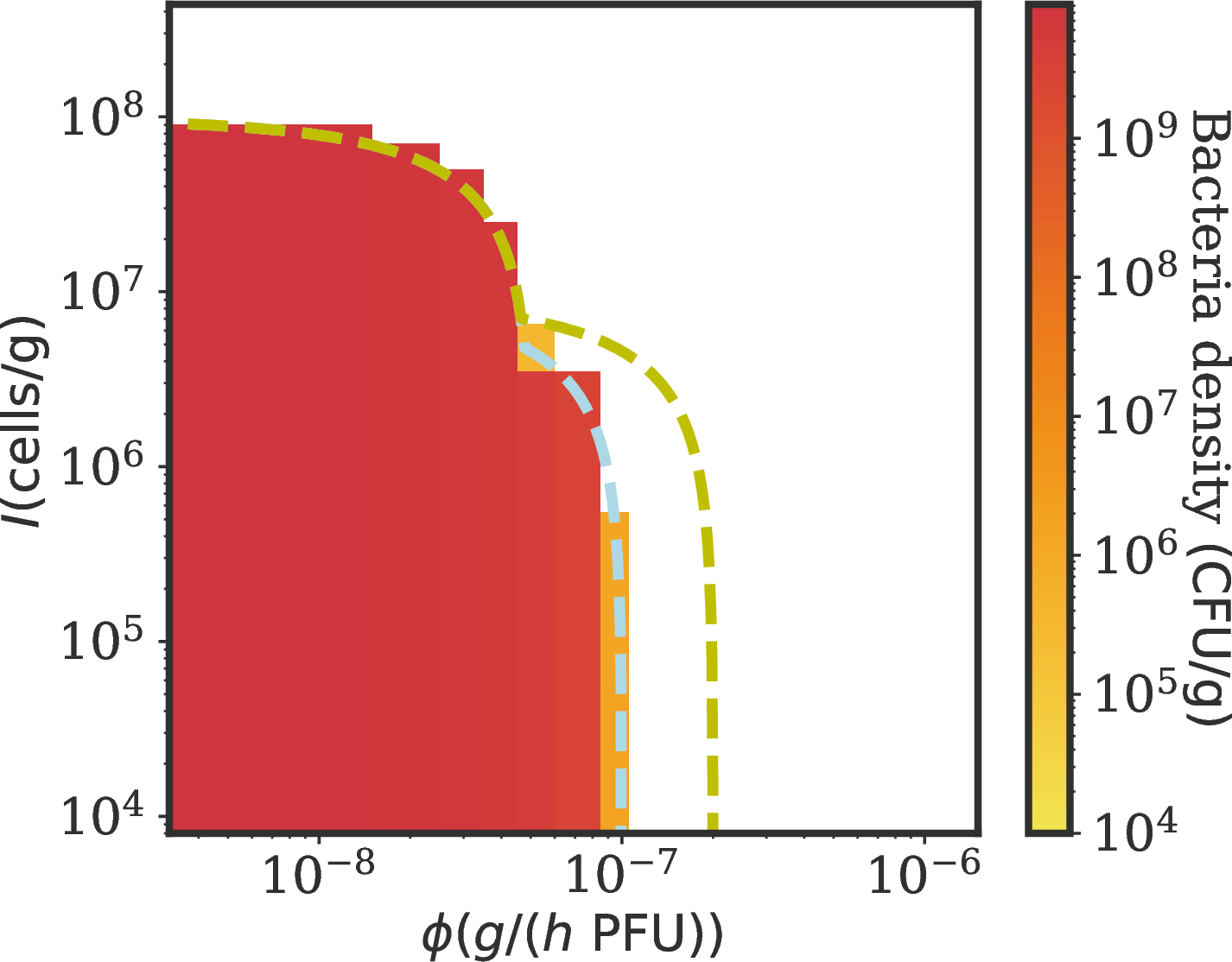

Supplement: S3 Fig — Numerical simulations of the model in Eq. (4) varying I and ϕ, with a single phage P2 and p = 0 . 5. The colormap represents the density of bacteria in the last part of the numerical simulations. In this extreme case, a single-phage treatment works better than two phages. The dashed lines compare Eq. (11) for p = 0 . 5 with two phages (yellow) with the transition for a single generalist phage (lightblue, equivalent to Eq. (11) with p = 1 ). Simulation parameters are reported in Table 2. (TIF) [file pcbi.1012793.s004.tif]

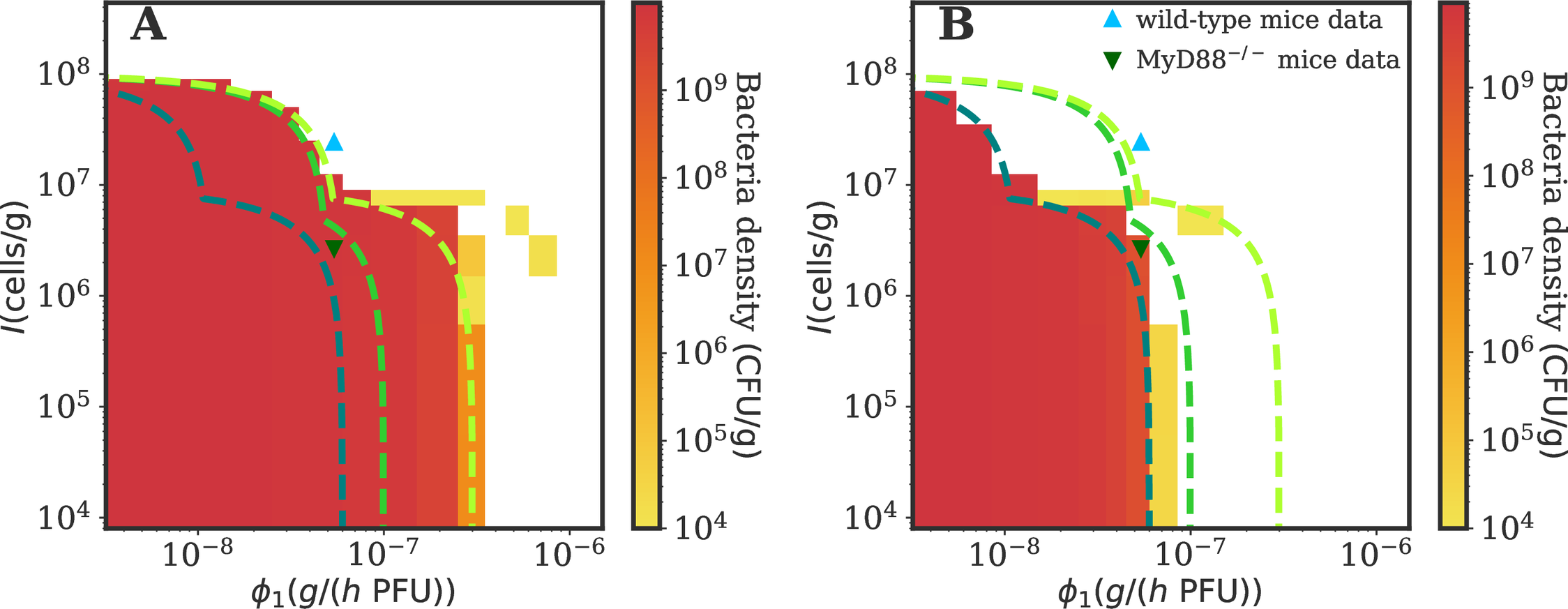

Supplement: S4 Fig — Numerical simulations of the model in Eq. (4) with ϕ2=σϕ1, varying I and ϕ1, for σ = 1 ∕ 5 (A) and σ = 5 (B). The color map represents the density of bacteria in the last part of the numerical simulations. A) While phage cocktails can still drive bacteria to extinction in immunocompromised hosts, a low σ can jeopardize the treatment outcome. B) A high σ improves therapy, especially in immunocompetent hosts. The dashed lines show Eq. (S19) for σ = 1 ∕ 5, 1 and 5 (lighter green, green, teal). The simulations agree reasonably well with the analytical approximation. The two triangle markers correspond to the I and ϕ inferred in [16] from in vivo experiments, for wild-type (immunocompetent, upward light-blue triangle) and MyD88-∕- (immunodeficient, downward dark-green triangle) mice. Simulation parameters are reported in Table 2. (TIF) [file pcbi.1012793.s005.tif]

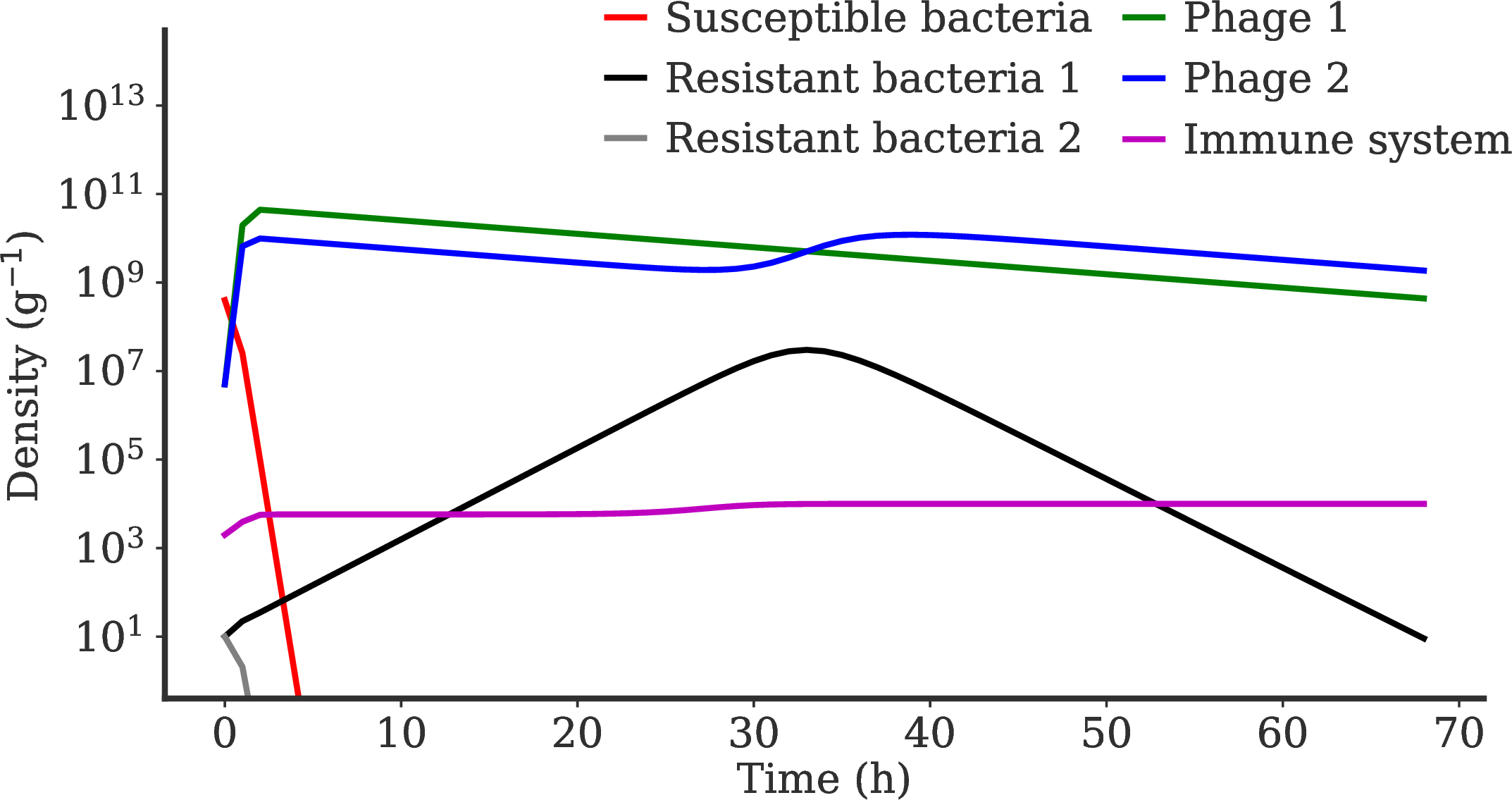

Supplement: S5 Fig — Numerical simulations of the model in Eq. (4) varying I and ϕ1, for σ = 1 ∕ 5 ϕ1=5⋅10-7 and I=104. The rest of the simulation parameters are reported in Table 2. (TIF) [file pcbi.1012793.s006.tif]
